# Event and Comment

**Published:** 1909-11-15

**Authors:** 


// DENTAL REGISTER.

Vol. LXIII. November 15, 1909. No. 11.

### EVENT AND COMMENT.

**LOVING CUP TO THE MILLER DENTAL CLUB.** At the close of the recent meeting of the 5th International Dental Congress, in Berlin, a pleasant incident was the presentation of a handsome silver loving cup to the Miller Dental club of Berlin, by the American delegates to the congress, as an expression of their appreciation of the innumerable courtesies extended to them by the club and its members. Dr. H. E. Hosley, of Springfield, Mass., made the presentation speech in which he took occasion to comment on the men who had made American dentistry famous in Germany and to commend the American contingent which was maintaining the American reputation in spite of the great strides made by the continental practitioners in recent years. Dr. N. S. Jenkins made some timely remarks in explanation of the origin and proper use of the loving cup. Dr. Henry W. Bodecker, as Vice President of the club, accepted the cup in the absence of Dr. Abbot, who had been called to America by the death of his niece, Dr. Miller's daughter, who died in Middletown, Connecticut, where her husband is Professor of Physics in Wesleyan University. On the same occasion the American dentists presented Dr. H. W. Bodecker, a silver mounted ink stand in appreciation of his many favors as chairman of the committee of reception for the American delegates. Dr. Bodecker as interpreter and reception committee was the right man for the place, and he did everything possible to care for the interests of the American delegates and was a very busy and much worked individual, and his services to all English speaking dentists were generously extended and most gratefully appreciated.

**BUSINESS BUILDING.** Under this heading our esteemed contemporary, the *Dental Digest*, conducts a department in which business methods are discussed. In the October issue a contributor from a small town in Indiana, states his experience and shows that he is not saving very much money although he has the best practice in the town of three thousand people, with a good farming country also to contribute. His total receipts for the year average about $2,000.00, and his office and living expenses nearly $1900.00 leaving him a yearly profit of a little over one hundred dollars. Certainly not a great prospect ahead at that rate, and we don't wonder that he views the future with some anxiety, especially, as he has a growing family that will become increasingly more expensive year by year. The dentist thinks he has exhausted the resources of his community and wants to know what he shall do, as he can't very well move to a larger town as he has no capital to meet expenses while building a new practice. We don't know the situation well enough to give a proper answer, perhaps, but we would venture a suggestion that comes to us from glancing at the items of his income. His largest income is derived from crown and bridge work, $560.00, next plate work, then amalgam fillings, gold fillings, treatments, extractions, and etc., to cleaning and polishing $77.00, inlays $32.00. It is easy to see what kind of a community this practitioner has to deal with and it may be impossible to change matters in such a community, but we are confident with a proper and determined effort this income could wit a little thought, be materially increased. We venture to say that among those 3000 people there are at least ten boys and ten girls between the ages of ten and sixteen that sadly need orthodontic treatment if the matter was properly put to the attention of their parents, and from two to five hundred dollars at least could by systematic effort along that line be added to that income. Then there was but $32.00 received from inlays, which at very low fees would mean only eight or ten inlays. Isn't there a better result possible in that direction, an income ten times greater, even in such a community, is not an impossibility. But the one item in the whole inventory that staggers us most is that which reports an average of $77.00 each year for cleaning and polishing. At the dentist's own rates of fees, which he schedules at from one to five dollars, or at the lowest rate there is an admission that he cleaned and polished only seventy-seven people's teeth in a year, or one and a half each week. Now does it seem reasonable to suppose that in a community of over 3000 people that only 77 such operations could be required in a year of time? From an experience of many years we are free to say that we have never seen a community where the people took such excellent care of their teeth that only one in forty of them required the help of a dentist each year to free his teeth from deposits which were threatening the health of his teeth and gums, and which he could not remove by the utmost care with the personal mouth toilet. It may be that the people in that village take exceptional care of their teeth and so do not need professional service. We are inclined to doubt any such suggestion, and venture to affirm that every man, woman and child in that community not only requires but would gladly pay at least one dollar each year for a satisfactory cleaning and polishing of his teeth. At one dollar each, this would boost our country dentist's income nearly $3000.00 at once, and he needn't make a plate crown or bridge or even extract a tooth or make an Amalgam filling and live as well as he now does, and have a thousand dollars in the bank at the close of the year instead of $114.00. Yea, even better than that, for this kind of work would involve no great outlay for materials or office equipment. But suppose he could charge $5.00 for each treatment, or could treat each patient five times each year, which would be little enough for their best interests, see what a boost in that income he would have, $15,000.00, or a net savings in the bank of $13,000.00 each year! Why my dear sir you have a regular bonanza in your own door yard and don't know it. Wake up and get busy with the most important work known to your profession. The people are really suffering for this very ministration which you are competent to give them. Read the third and fourth chapters of the book of Jonah and pray that the Lord may turn your eyes and thoughts towards Ninevah,—I don't know as that is the exact name of your town,—but come out from the shadow of the gourd vine and hearken to the Divine call to duty, and cease to complain because the Lord doesn't prosper you as you think you deserve. Get busy with the real work and your fears of the future will vanish.

